# *Ex vivo* modulation of intact tumor fragments with anti-PD-1 and anti-CTLA-4 influences the expansion and specificity of tumor-infiltrating lymphocytes

**DOI:** 10.3389/fimmu.2023.1180997

**Published:** 2023-06-08

**Authors:** Thomas Morgan Hulen, Christina Friese, Nikolaj Pagh Kristensen, Joachim Stoltenborg Granhøj, Troels Holz Borch, Marlies J. W. Peeters, Marco Donia, Mads Hald Andersen, Sine Reker Hadrup, Inge Marie Svane, Özcan Met

**Affiliations:** ^1^ National Center for Cancer Immune Therapy (CCIT-DK), Department of Oncology, Copenhagen University Hospital, Herlev, Denmark; ^2^ Department of Health Technology, Technical University of Denmark, Lyngby, Denmark

**Keywords:** tumor-infiltrating lymphocyte (TIL), checkpoint inhibition, metastatic melanoma, cancer immunotherapy, tumor microenevironment, adoptive cell immunotherapy, DNA Barcoding

## Abstract

Checkpoint inhibition (CPI) therapy and adoptive cell therapy with autologous tumor-infiltrating lymphocytes (TIL-based ACT) are the two most effective immunotherapies for the treatment of metastatic melanoma. While CPI has been the dominating therapy in the past decade, TIL-based ACT is beneficial for individuals even after progression on previous immunotherapies. Given that notable differences in response have been made when used as a subsequent treatment, we investigated how the qualities of TILs changed when the *ex vivo* microenvironment of intact tumor fragments were modulated with checkpoint inhibitors targeting programmed death receptor 1 (PD-1) and cytotoxic T-lymphocyte-associated protein 4 (CTLA-4). Initially, we show that unmodified TILs from CPI-resistant individuals can be produced, are overwhelmingly terminally differentiated, and are capable of responding to tumor. We then investigate these properties in *ex vivo* checkpoint modulated TILs finding that that they retain these qualities. Lastly, we confirmed the specificity of the TILs to the highest responding tumor antigens, and identified this reactivity resides largely in CD39^+^CD69^+^ terminally differentiated populations. Overall, we found that anti-PD-1 will alter the proliferative capacity while anti-CTLA4 will influence breadth of antigen specificity.

## 1 Introduction

Adoptive cell therapy using autologous tumor-infiltrating lymphocytes (TIL-based ACT) has been clinically explored for the treatment of metastatic melanoma for decades (1–4). This was the most effective therapeutic strategy until the inception of checkpoint inhibition (CPI) therapy which drastically changed the treatment landscape of melanoma cancers (5). Monoclonal antibodies targeting programmed death receptor 1 (PD-1) and cytotoxic T-lymphocyte-associated protein 4 (CTLA-4) became nearly universal first-line therapy (6, 7). While response rates of up to 58% were reached when used in combination, patients experience extensive toxicities frequently followed by relapse (5). Additionally, some studies indicate a negative impact on subsequent TIL-based ACT (3, 8). While this evidence suggests that immune modulation with CPI may impair the likelihood of positive outcome for the cell therapy, we have shown that individuals with recurrent disease after anti-PD-1 treatment can still produce TILs capable of eradicating melanoma and mediate clinical response (9). Similarly, we have shown that individuals who are resistant to multiple lines of CPI can still produce functional TILs (10, 11).

PD-1 and CTLA-4 interactions with their natural ligands, PD-L1 and CD80/CD86, respectively, promote tumor growth through multiple mechanisms including T cell anergy and apoptosis (12, 13). These interactions are also known to inhibit T cells through metabolic influence (14). Glycolysis is essential for execution of effector mechanisms as well as cell proliferation and differentiation (15, 16). In contrast, PD-1 ligation inhibits the switch from oxidative phosphorylation to glycolysis, which is essential for T cell effector function (17). Additionally, the overlap between tumor reactivity and exhausted phenotype is well established (18, 19). A recent report by Krishna, et al. has illustrated the importance of stem-like populations, characterized by a CD39^-^CD69^-^ phenotype, for clinical response (20). While CD39^-^ populations appear vital for TIL-based ACT, recent evidence has shown that patients who respond to anti-PD-1 therapy have high infiltration of CD39^+^ resident memory T cells (21). While there does appear to be a phenotypic CD39/PD-1 axis, Gangaev et al. have shown that the scope of antigen reactivity can be broadened by anti-CTLA-4 treatment but not anti-PD-1 treatment, in the case of metastatic melanoma (22). Through *ex vivo* modulation of the tumor microenvironment with CTLA-4 blocking antibodies, our group has shown similar broadening of the tumor-reactivity within the CD8^+^ compartment of TILs in ovarian cancer (23). CPI has been incorporated with tumor fragments in multiple similar studies to influence downstream effects of TILs (24–26).

Given the detrimental effects immunological checkpoints can have on the cellular immune response to cancer, we sought to investigate how disruption of checkpoint interactions would affect these qualities in melanoma TILs. With tumor material obtained from individuals with resistance to checkpoint therapy enrolled in clinical trials at our center, we altered the tumor microenvironment (TME) of intact tumor fragments by incorporating anti-CTLA-4 or anti-PD-1 monoclonal antibodies directly into the culture medium and subsequently harvesting the outgrown TILs. We evaluated the various phenotypic, metabolic, and functional qualities of the cells using a wide range of conventional and novel methods.

## 2 Results

### 2.1 Profiling of TILs derived from immunotherapy-resistant melanoma patients

**Figure 1:**
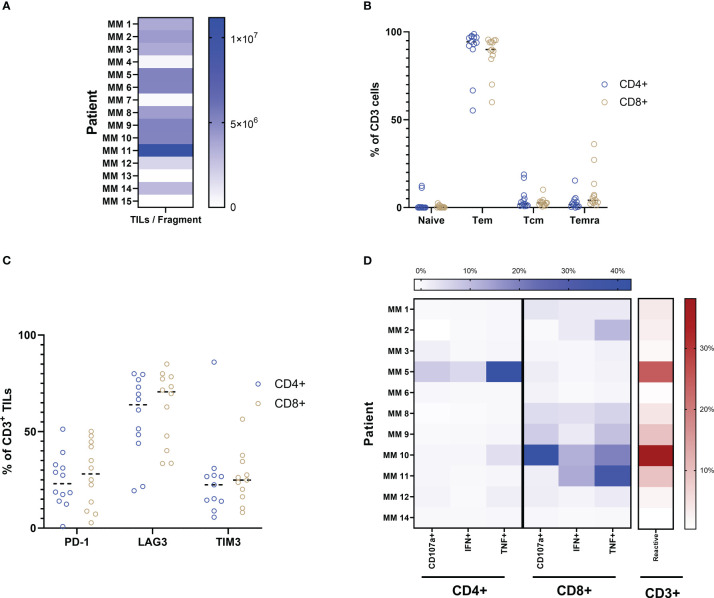
Production and characterization of yTILs. **(A)** TIL cultures were established from 11 out of 15 patient samples. **(B)** Both CD4+ and CD8+ TILs are primarily of the Tem phenotype. Exhaustion markers PD-1, LAG3 and TIM3 are expressed to some degree in almost all cultures **(C)**. ICS analysis is shown as a heatmap in **(D)**. Color intensity increases with percent of positive frequency of parameter within CD4 or CD8 population shown in blue. The heatmap to the right shows the percent of total reactive cells within the CD3 population indicated in red. Calculation for reactivity is described in Methods section.

This study included samples from 15 patients from three clinical trials conducted at our center (9–11). All patients included in these trials have developed resistance to at least one line of CPI therapy (**Table 1**). We initially sought to better understand the phenotypic profile and functional abilities of TILs generated from tumor fragments of CPI-resistant individuals. Through conventional production methods using high-dose IL-2 as previously described (10, 11), minimally cultured TILs were successfully established from 11 out of the 15 total patient samples obtained. TIL cultures were considered established if greater that 1 x 10^6^ TILs were produced per fragment, with eight fragments per condition. **Figure 1A** shows the spectrum of TIL-outgrowth, with fragments from patient 11 the most productive with 11 x 10^6^ TILs/fragment, and patient 12 least productive with 1.7 x 10^6^ TILs/fragment. Cultures were unable to be established from clinical samples from patients 4, 7, 13 and 15 and were excluded from further analyses. Flow cytometry was used for phenotype characterization of TILs. Effector-memory (Tem) and naïve T cells, respectively, made up the most and least abundant phenotypes across all patient samples in both CD4^+^ and CD8^+^ subsets, as shown in **Figure 1B**; **Table 2** displays individual marker frequency. The extent of exhaustion was assessed by flow cytometry using the parameters PD-1, LAG3 and TIM3 displayed in **Figure 1C** with CD8^+^ TILs having higher median expression of all three markers. Reactive abilities of the TILs were then tested by tumor-stimulation followed by intracellular staining (ICS) and flow cytometry. Autologous tumor was prioritized for stimulation experiments. When unavailable, allogenic tumor was used (**Table 2** shows tumor pairing). As shown in **Figure 1D**, the reactivity profile of TILs varies greatly between samples. CD8^+^ TILs showed to be overwhelmingly more reactive to tumor, with the exception of TILs produced from patient 5. Additionally, the bulk of the total reactivity came from tumor necrosis factor (TNF) production, except for TILs from patient 10, which was highly CD107a^+^ in the CD8^+^ compartment. Overall, 73% of tumor fragments produced established TIL cultures. Of these, measurable reactivity was detected upon tumor stimulation from all samples, showing that TILs from immunotherapy-resistant melanoma patients can still display anti-tumor reactivity.

**Table 1:** Patient profile. Detail of patients from whom tumor samples were obtained.

Patient #	Age	Sex	HLA Type	ECOG PS	Primary tumor origin	Biopsy site	Stage at tumor excision	Previous treatment
MM 1	52	M	A1	0	Skin	SC	IIIC	pem
MM 2	55	M	A2, A3	0	Unknown	LN	M1b	ipi, pem
MM 3	60	F	A2	0	Unknown	LN	M1c	ipi, pem
MM 4	64	F	A2, A3	0	Skin	SC	M1c	ipi, pem
MM 5	55	M	A2	1	Skin	SC	M1c	ipi
MM 6	53	M	A2, A3	1	Skin	SC	M1c	ipi, nivo
MM 7	50	F	A1, A2	0	Skin	LN	M1b	pem
MM 8	59	F	A1	1	Unknown	SC	M1c	ipi, pem
MM 9	52	F	A1	0	Skin	Lung	M1c	pem
MM 10	73	M	A3, A11	0	Unknown	SC	M1c	ipi, pem, nivo+rela
MM 11	67	F	A2, A3	0	Skin	SC/LN	M1c	ipi, pem
MM 12	54	M	A2	0	Skin	SC	M1a	pem
MM 13	53	M	A2	1	Skin	SC	M1c	pem
MM 14	26	M	A1, A24	0	Skin	LN	M1c	pem
MM 15	70	F	A2	1	Skin	LN	M1c	pem, dabra+tram, ipi+nivo

SC, subcutaneous; LN, lymph node; Pem, Pembrolizumab (anti-PD-1); Ipi, Ipilimumab (anti-CTLA-4); Nivo, Nivolumab (anti-PD-1); Rela, Relatlimab (anti-LAG-3); Dabra, Dabrafenib (BRAF-inhibitor); Tram, Trametinib (MEK-inhibitor); ECOG PS, Eastern Coorperation Oncology Group Performance Status.

**Table 2:** Sample overview including TILs/fragment, CD4 and CD8 frequencies, and ICS tumor stimulation for the three TME conditions.

Patient #	CPI Modulation	TILs/Fragment (x10^6)	%CD4+ of CD3+	%CD4+ of CD3+	ICS Tumor Stimulation
MM 1	Alone	3,66	31,2	66,2	Allogenic
	Anti-CTLA-4	2,63	29,2	65,7	
	Anti-PD-1	2	15,5	80,6	
MM 2	Alone	4,23	47,1	47,3	Allogenic
	Anti-CTLA-4	4,23	58,1	37,2	
	Anti-PD-1	6,21	70,8	25,1	
MM 3	Alone	3,6	37,7	48,8	Allogenic
	Anti-CTLA-4	7,43	26,7	63,2	
	Anti-PD-1	3,93	31,0	24,4	
MM 4	Alone	0,5	27,5	69,3	Allogenic
	Anti-CTLA-4	0,74	39,4	56,1	
	Anti-PD-1	8	7,4	91,0
MM 5	Alone	5,18	52,0	40,7	Autologous
	Anti-CTLA-4	2,78	87,2	10,8	
	Anti-PD-1	3,65	61,6	35,1	
MM 6	Alone	5,14	87,9	11,1	Allogenic
	Anti-CTLA-4	2,6	7,9	83,7	
	Anti-PD-1	4,3	48,6	49,3	
MM 7	Alone	0,23	48,1	47,4	Allogenic
	Anti-CTLA-4	0,09	46,0	44,9	
	Anti-PD-1	0,95	22,6	68,4	
MM 8	Alone	4,15	21,6	75,6	Allogenic
	Anti-CTLA-4	2,73	72,9	24,9	
	Anti-PD-1	4,35	26,8	69,9	
MM 9	Alone	5,25	18,7	79,6	Autologous
	Anti-CTLA-4	2,55	11,8	84,9	
	Anti-PD-1	2,7	33,2	63,0	
MM 10	Alone	5,16	8,9	89,7	Autologous
	Anti-CTLA-4	6,01	12,7	85,9	
	Anti-PD-1	6,23	11,0	87,4	
MM 11	Alone	11,18	69,1	29,3	Autologous
	Anti-CTLA-4	1,78	35,3	56,0	
	Anti-PD-1	16,2	2,0	97,1	
MM 12	Alone	1,76	28,0	68,0	Autologous
	Anti-CTLA-4	3,44	9,8	83,0	
	Anti-PD-1	5,41	17,2	77,5	
MM 13	Alone	0,03	57,2	33,2	n.d.
	Anti-CTLA-4	0,3	23,8	69,3	
	Anti-PD-1	17,43	6,7	91,9	
MM 14	Alone	2,98	39,2	59,2	Allogenic
	Anti-CTLA-4	2,69	50,5	43,6	
	Anti-PD-1	6,94	78,2	19,1	
MM 15	Alone	n.d.	n.d.	n.d.	n.d.
	Anti-CTLA-4	n.d.	n.d.	n.d.	
	Anti-PD-1	n.d.	n.d.	n.d.	

Red text indicates culture not established. N.d. indicates no data due to lack of TIL production.

### 2.2 Profiling of TILs derived from CPI modulated tumor fragments

**Figure 2:**
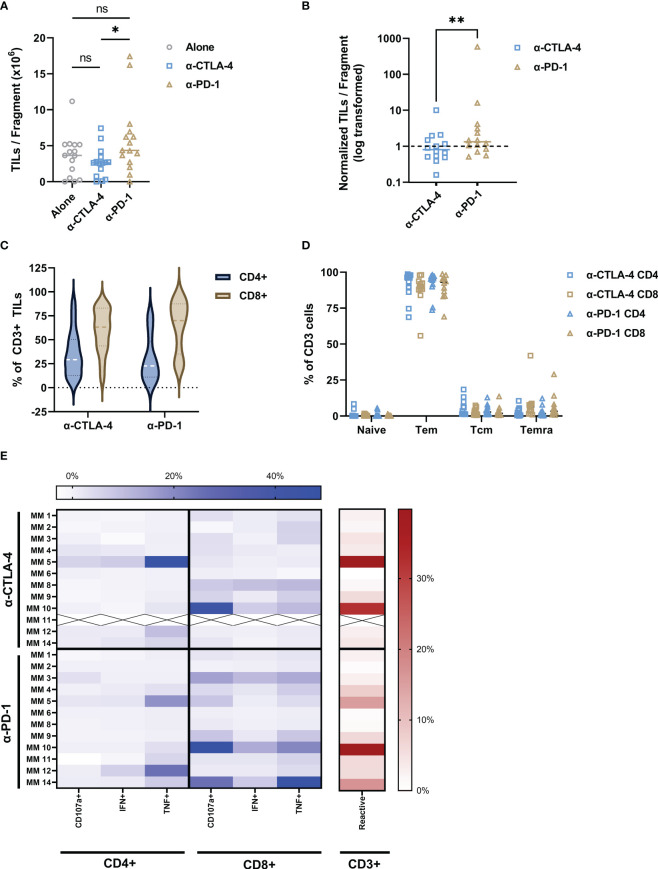
Addition of CPI to intact tumor fragments produce tumor-reactive TILs. **(A)** Anti-CTLA-4 and anti-PD-1 addition to the TME resulted in production of TILs with a median of 2.66 x 10^6^ and 5.88 x 10^6^ TILs/fragment, respectively with statistical difference between the two groups (P value = 0.0107). TILs alone dataset included as reference. **(B)** The number of TILs per fragment for both anti-CTLA-4 and anti-PD-1 conditions were normalized to the TILs alone for each patient. Data points indicate change in number of TILs per fragment from TILs alone baseline (dashed line). PD-1 inhibition produces significantly more TILs per fragment than anti-CTLA-4 (P value = 0.0085). **(C)** Both CPI conditions produced high CD8:CD4 ratios and were dominantly Tem **(D)**. ICS analysis is shown as a heatmap in **(E)**. Color intensity increases with percent of positive frequency of parameter within CD4 or CD8 population shown in blue. The heatmap to the right show the percent of total reactive cells within the CD3 population in red. “X” indicates sample unavailable. Calculations for normalization and reactivity are described in Methods section. *p < 0.05; **p < 0.001. Exact p values are listed in image description.

Although all samples were obtained from CPI-treated and -resistant melanoma patients, we further explored the influence of CPI introduction directly to the intact tumor fragment. **Figure 2A** shows the establishment and proliferative ability of TIL cultures form patient-derived samples. Anti-CTLA-4 TIL cultures were established in 11 out of 15 samples, while anti-PD-1 cultures were established in 14 out of 15. While the total TILs per fragment were significantly higher with the addition of PD-1-blocking antibodies compared to that of CTLA-4 (P value = 0.0107), addition of checkpoint inhibitors did not statistically improve overall TILs per fragment compared to TILs alone (anti-CTLA-4, P value = 0.3054; anti-PD-1, P value = 0.1040). **Figure 2B** shows TILs per fragment from both CPI groups normalized to the alone group for each patient as detailed in section 4.2. Anti-PD-1 modulation produced significantly more TILs per fragment than that of anti-CTLA-4 when normalized to TILs from no checkpoint exposure (P value = 0.0085. Anti-CTLA-4 average normalized count: 1.57, +/-SD: 2.4; Anti-PD-1 average normalized count: 43.96, +/-SD: 149.0). **Figure 2C** shows that the bulk of TILs from all samples resides in the CD8^+^ compartment, while Tem is the dominant phenotype in both CD4^+^ and CD8^+^ subsets (**Figure 2D**). These data can be found in **Table 2**. The expression of exhaustion markers LAG3, TIM3 and PD-1 were not influenced by CPI (**Supplementary Figure S1**). Further, naïve cells are the least abundant phenotype across the patient samples. The addition of anti-CTLA-4 and anti-PD-1 into the TME produced TILs that were able to react to tumor as measured by ICS as shown in **Figure 2E**. Modulation of the TME with anti-CTLA-4 and anti-PD-1 similarly produced the most reactive TIL from patients 5 and 10, although they have distinct expression profiles. TILs derived from patient 6 were evidently non-reactive whether the TME was modulated with anti-CTLA-4 or anti-PD-1. Overall, CD8^+^ subsets demonstrated the most anti-tumor reactivity. These results show that TIL cultures can be established after disrupting either CTLA-4 or PD-1 interactions in the TME of CPI-resistant tumors, and that they are capable of anti-tumor responses.

Since the addition of anti-PD-1 to the tumor fragments produced established TIL cultures from three more patients than either the alone or anti-CTLA-4 conditions, we investigated how PD-1 interference would affect the TILs in rapid expansion protocol (REP). TILs from the previous section, and TILs from anti-PD-1 modulated tumor fragments were expanded *via* REP as previously described (10, 11) with or without the addition of 5 g/mL anti-PD-1 supplemented in the culture medium. While the difference in REP yield was variable between patients, alteration of the REP environment with anti-PD-1 went largely unchanged (**Supplementary Figure S2**).

### 2.3 Metabolic characteristics of TILs remain unchanged with CPI-modulation

**Figure 3:**
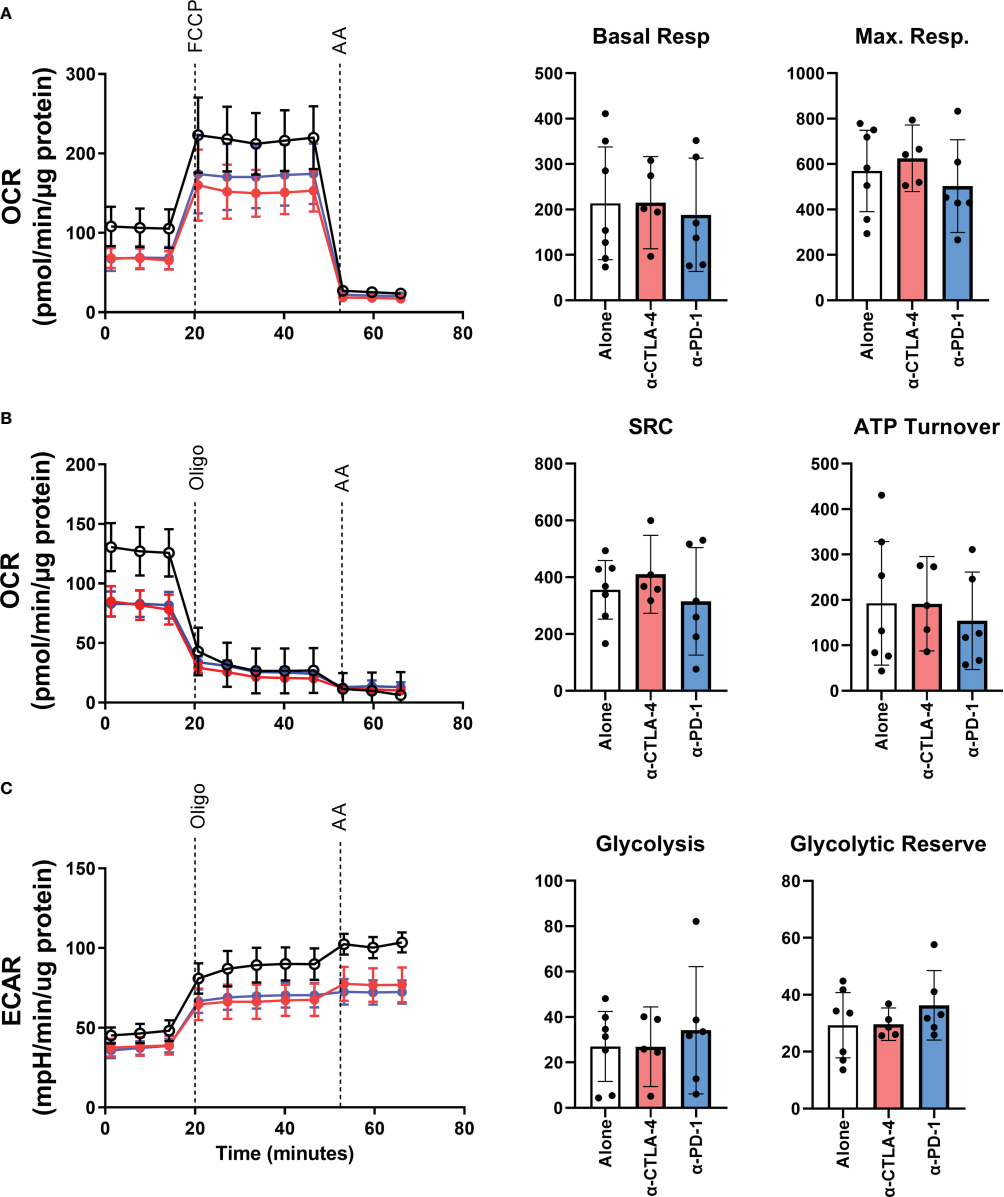
The bioenergetic effects of CPI treatment on patient-derived YTILs. **(A–C)** Line plots in show representative example of oxygen consumption rate (OCR) and extracellular acidification rate (ECAR) from Patient MM 3. Time of oligomycin, FCCP, and antimycin injections are marked in the figures. Bar graphs in **(A)**, **(B)** show basal respiration, maximal respiration, spare respiratory capacity and ATP turnover for each CPI-treated condition. Bar graphs in **(C)** show glycolysis and glycolytic reserve. The Mann-Whitney U test was used to compare sets of unpaired data. We observed no statistically significant differences between the CPI-treated groups. OCR, oxygen consumption rate; ECAR, extracellular acidification rate; Oligo, oligomycin; FCCP, carbonyl cyanide-p-trifluoromethoxyphenylhydrazone; AA, antimycin A; SRC, spare respiratory capacity.

Interactions with immunological checkpoints have been shown to have downstream metabolic effects (14). Therefore, we sought to investigate if checkpoint inhibition in the TME would alter the metabolic qualities of TILs. The bioenergetics of patient-derived TILs was measured in real-time using an XF-96 Extracellular Flux Analyzer measuring oxygen consumption rate (OCR) and extracellular acidification rate (ECAR). Line graphs in **Figures 3A–C** show a representative example of OCR and ECAR measurements for patient derived TILs across the three TME conditions. As summarized in the bar graphs of **Figures 3A, B**, interfering with checkpoint interactions within the TME does not alter the oxidative phosphorylation profile of TILs as measured by basal or maximum respiration, spare respiratory capacity, nor ATP turnover. Similarly, glycolytic abilities of the TILs were retained regardless of TME intervention as shown in the bar graphs of **Figure 3C**.

### 2.4 TME-modulation with anti-CTLA-4 broadens the tumor specificity of TILs

Profiling of peripheral T cells has shown that systemic treatment with CPI can influence the scope of antigen reactivity (22). Therefore, we sought to investigate the effect local CPI treatment of intact tumor fragments would have on TILs in the TME. The specificity was assessed using antigen libraries consisting of 156 HLA-A*02:01 restricted tumor antigens and a virus-derived peptide library comprised of 9 peptides. Peptide : HLA complexes were assembled on dextran-multimers coupled to unique DNA barcodes that corresponded to each peptide (27, 28). Tumor-associated multimers and virus-associated multimers were coupled to PE and APC fluorophores, respectively. This DNA barcoding allows for staining of TILs from HLA-A2-positive individuals with multimer pools, FACS sorting and subsequent amplicon sequencing to match with and identify epitope specificity of the TIL populations. **Supplementary Figure S3** shows a representative plot of sorted tetramer-positive populations. After amplicon sequencing, specificities could be identified, and the sum of the estimated frequencies (sum est feq) could be quantified.

**Figure 4:**
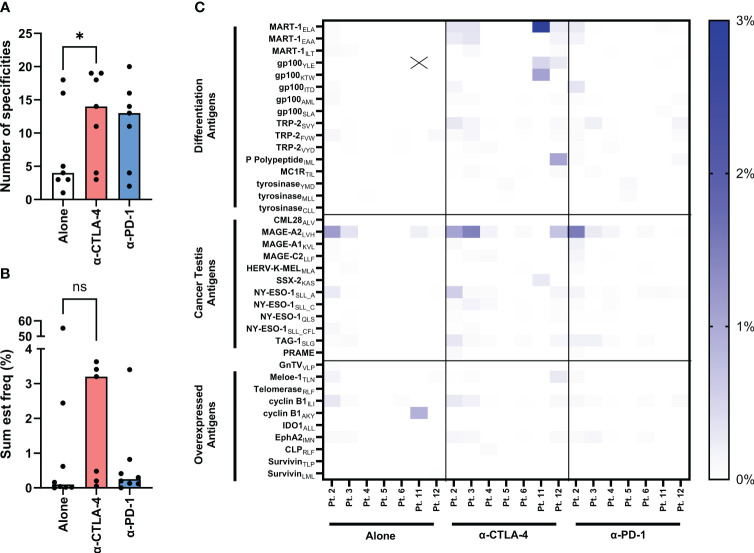
Evaluation of tumor-specificities of yTILs produced with IL-2 alone or CPI. yTILs were stained with pooled tetramers loaded with 156 unique HLA-A2-restricted epitopes. **(A)** shows the number of tumor antigens yTILs from each patient had across conditions with anti-CTLA-4 group having statistically significant more specificities than alone (P value = 0.0156), while the anti-PD-1 group is not significant (P value = 0.938). **(B)** shows the sum of the estimated frequencies of tumor antigens across conditions. The sum est freq of the anti-CTLA-4 group is not statistically significant (P value = 0.2969). The individual specificity frequencies for each tumor antigen across 3 antigen categories are represented with a heatmap **(C)**. “X” indicates value of 54.17%. Wilcoxon test was applied to compare paired data sets. “ns” = not significant; “*” represents the p value which is listed in the image description.

**Figure 4A** shows the number of tumor specificities recognized by TILs from each TME condition. The influence of CPI in the intact fragments increases the antigen specificities of both checkpoint conditions. The averages and standard deviations are 7.14 (+/- 6.4), 12.57 (+/- 6.3), 11.43 (+/- 5.9) for the conditions alone, anti-CTLA-4 and anti-PD-1, respectively. This is statistically significant with the addition of CTLA-4 blockade (P value = 0.0156) but not with the addition of anti-PD-1 (P value = 0.0938). This is reflected in the sum est freq of tumor specific TILs (**Figure 4B**) in the anti-CTLA-4 group in which the sum est freq is increased in all CTLA-4 inhibited samples, although it is not statistically significant (P value = 0.2969). The averages and standard deviations are 58.59 (+/- 18.14), 15.73 (+/- 1.79), 5.38 (+/- 1.06) for the conditions alone, anti-CTLA-4 and anti-PD-1, respectively. However, this is not true of the TILs from patient 11 as the unmodulated TILs from this individual show a sum est freq of tumor-reactivity that is 10- to 100-fold greater than that of any other sample. The top 20 specificities in all of the TILs are shown in **Supplementary Figures S4** and the top 20 sum est freq is shown in S5. The heatmap in **Figure 4C** depicts the individual estimated frequency of tumor antigens for each patient within the three TME conditions. Here, it can be seen that the bulk of the sum est freq from patient 11 as noted above comes from specificity towards the gp100_YLE_ peptide. Antigens with no detected reactivity are not shown, resulting in 38 tumor antigens across three categories: differentiation antigens, cancer testes antigens, and overexpressed antigens. Modulation with CPI had no effect on the number and sum est freq of virus specificities of TILs (**Supplementary Figures S6, 7**, respectively). **Supplementary Table S1** displays the full list of antigens.

### 2.5 TILs derived from CPI-modulated tumor fragments retain antigen-experienced phenotype within tumor specific populations

**Figure 5:**
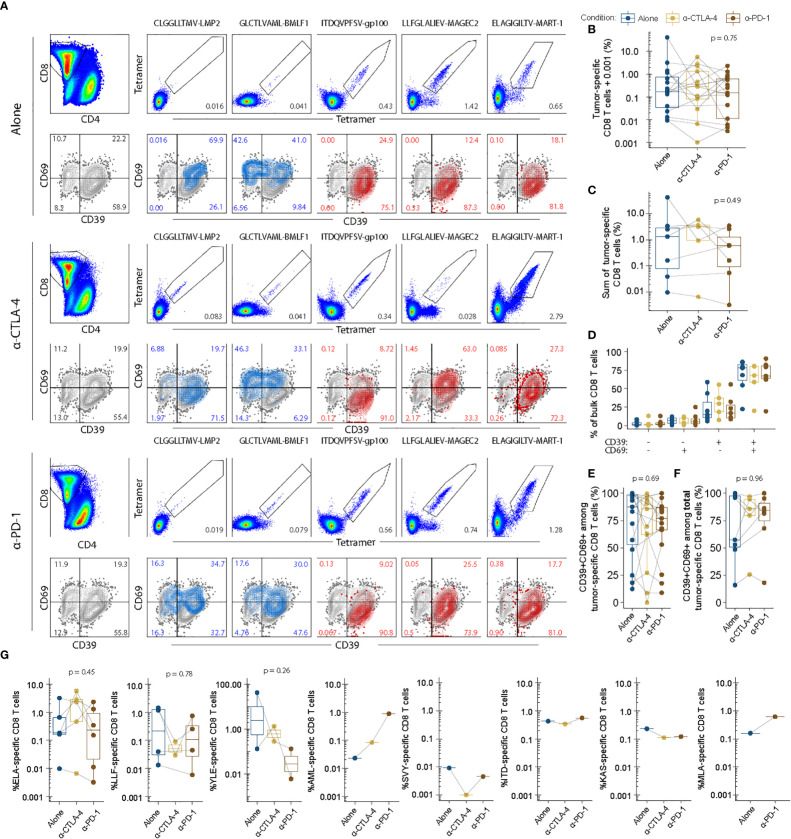
Tumor-specific recognition and phenotype is largely unchanged between expansion conditions. Epitope-specific recognition of tumor-shared antigens numerated with fluorescent combinatorial encoding of pMHC tetramers. All epitopes were restricted to HLA-A*02:01. Protein expression of CD69 and CD39 in bulk CD8 T cells overlaid by data from each specific population as shown in **(A)**. Enumeration of tumor-specific ie tetramer double positive CD8 T cells **(B)**. All populations for each donor are plotted individually. **(C)** Enumeration of total tumor-specific CD8 T cells i.e. the sum of all individual tetramer double positive CD8 T cells per donor and expansion condition. **(D)** Stem-like and activated phenotypes for bulk CD8 T cells for each condition of interested described previously (20). **(E)** Terminally differentiated phenotypes among tumor-specific CD8 T cells. **(F)** Terminally differentiated phenotypes among total tumor-specific CD8 T cells. **(G)** Individual enumeration of each tumor-specific population of interest for each condition with each dot representing individual donors and their responses. ELA, ELAGIGILTV-MART-1. LLF, LLFGLALIEV-MAGEC2. YLE, YLEPGPVTA-gp100. AML, AMLGTHTMEV-gp100. SVY, SVYDFFVWL-TRP-2. ITD, ITDQVPFSV-gp100. KAS, KASEKIFYV-SSX-2. MLA, MLAVISCAV-HERV-K. P values were evaluated using unpaired kruskal-wallis test.

Based on the initial screens using barcode-labelled pMHC dextran multimers, we verified a number of tumor-specific populations of CD8 T cells recognizing tumor-shared antigens in context of HLA-A*02:01. The tumor antigens with the highest sum est freq were selected to further explore the impact CPI modulation had on TIL epitope specificity. While select tetramers failed due to strong pellet formation during staining (see methods), we found 46 populations across 16 TIL groups including 2 virus-specific populations of CD8 T cells. To avoid noise from single events, the lower threshold for detection was set at 0.001%. The frequency range above the threshold included responses from 0.0016% specific CD8 T cells to 41.7% gp100-specific CD8 T cells. Other noticeable antigens recognized included MAGE-C3, TRP-2 and MART-1, as well as TILs from a single patient recognizing synovial sarcoma X breakpoint protein 2 (SSX-2) and human endogenous retrovirus K. These data can be found in **Supplementary Figures S8.1 – S8.3**. **Figure 5A** shows the analysis of virus or tumor specificity overlaid with CD39 and CD69 expression. Frequency of tumor-specific CD8 T cells were highly variable and non-significantly changed between conditions suggesting that anti-CTLA-4 and anti-PD-1 did not exert strong positive or negative effects on expansion of antigen-specific CD8 T cells shown in **Figures 5B, C**.

The expression profile of CD39 and CD69 within the bulk of the CD8^+^ population remained unchanged with CPI TME modulation (**Figure 5D**). Within the tumor antigen specific CD8^+^ T cells, the percentage of CD39 and CD69 double positive cells varies greatly between patients. CPI modulation does not appear to alter this phenotype within each patient whether viewed as individual populations of tumor reactive CD8^+^ T cells (**Figure 5E**), or as the sum of total tumor reactivity (**Figure 5F**). **Figure 5G** depicts the heterogeneity of tumor specificities between patients and conditions.

Interestingly, we observed strongly differing frequencies of CD8^+^ T cells recognizing the same tumor epitope between expansion conditions within the same donor. This pattern appeared somewhat dynamic, whereby a few antigen-specificities would preferentially expand in each culture. In the donor presented in **Figure 5A**, MART-1-specific CD8 T cells for example appeared more expanded in the anti-CTLA-4 and anti-PD-1 conditions than the corresponding unmodulated condition (IL-2: 0.65%, Anti-CTLA4: 2.79%, PD-1: 1.28%). However, the unmodulated TILs instead contained a relatively higher frequency of MAGE-C2 specific CD8 T cells (IL-2: 1.42%, anti-CTLA-4: 0.028%, anti-PD-1: versus 0.74%). Similarly, gp100 were highly expanded in another donor during the IL-2 culture condition (41.7%) but underwent next to no expansion in anti-CTLA-4 (0.28%) and anti-PD-1 (0.13%) conditions. Given the similarity between the unmodulated and checkpoint-modulated conditions, such donor-specific variability is likely due to a high variability inherent in the expansion of TILs.

## 3 Discussion

TIL-based ACT has historically shown to be one of the most effective therapeutic options for patients with metastatic melanoma (3, 9). While the potential for successful outcome has been shown to decrease after other immunotherapeutic interventions prior to TIL-based ACT, this treatment is still a productive therapy for many patients (8, 10, 11). The incorporation of anti-PD1 and anti-CTLA-4 into the TME of intact tumor fragments had mixed overall effects on the TILs. Regardless of checkpoint inhibitor, some qualities of un-altered TILs were retained, such as the bioenergetic abilities and exhaustive phenotype. However, disruption of PD-1 interactions appeared to have proliferative effects on the TILs, while the effects of CTLA-4 inhibition were observed in their improved specificity. These changes as a result of modulation of the tumor fragment were narrow, which may have been a result of previous exposure to prior immunotherapies.

We were unable to establish TIL cultures from patients 7, 13, and 15 from tumor fragments that were unaltered, or with anti-CTLA-4. Anti-PD-1 rescued the establishment of TIL cultures in patients 7 and 13. This recovery and the improvement in total TIL production compared to anti-CTLA-4 are evident, given the well-known effects of PD-1 ligation on T cell apoptosis and proliferation (12, 29, 30). CTLA-4 interactions occur mostly during the priming phase of the immune response (31, 32). Perhaps the tumor reactivity we appreciated, measured by total number of specificities and sum est freq, is enriched with anti-CTLA-4 treatment because more tumor reactive TILs will receive the costimulation needed in the priming phase to become activated and localize to the tumor, thus boosting the T cell repertoire we found in these samples. Unmodulated TILs from patient 11 showed a sum est freq of tumor reactivity that was strikingly different from those derived from any other individual. This detected specificity was almost entirely due to the gp100yle peptide. gp100 is an immunogenic protein shown to elicit strong t cell responses (33, 34) and encodes the well-known tumor-associated antigen pmel17, which is a historical immunotherapeutic target of multiple past and ongoing clinical trials (35). Other experiments showed that CPI modulation had striking differences on all parameters for this patient. While the unmodulated and anti-PD-1 modulated samples were among the most prolific cultures when producing the TILs (11.2 x 10^6^ and 16.2 x 10^6^ TILs per fragment, respectively), CTLA-4 inhibition resulted in only 1.8 x 10^6^ TIL per fragment. Further, the CD4:CD8 ratio in the unmodulated TILs favorable towards CD4 with only 29.3% CD8^+^. Anti-CTLA-4 treatment changed this to 56% CD8^+^ while anti-PD-1 treatment drastically altered the CD8 compartment with 97.1% CD8^+^, which was the highest density in the dataset. Unfortunately, not enough anti-CTLA-4 TILs were obtained for other experiments.

All patient samples consistently displayed an antigen experienced Tem phenotype indicating that they are terminally differentiated even before undergoing the rapid expansion protocol, which is the second expansion phase of TIL-production for clinical use. This was uninfluenced by checkpoint modulation of the TME. CD39 is a late activation marker following antigen-specific stimulation and a marker for tumor-reactivity in the tumor microenvironment (36). Furthermore, CD39 is a known feature of exhausted CD8 T cells with immunosuppressive capacity (37). CD69 encodes a C-type lectin that functionally inhibits sphingosine-1-phosphate receptors (S1P1-5), which is associated with retention of CD8 T cells in lymphoid organs (38). Furthermore, CD69 represents a marker of tissue resident CD8 T cells (39) and is an early activation marker following TCR-dependent activation (40). As shown by Krishna and colleagues (20), the co-expression of CD39 and CD69 is a useful surrogate of terminal differentiation in expanded TIL cultures, where CD39^+^CD69^+^ are incapable of further proliferation. CD39 and CD69 double negative cells are correspondingly capable of renewing all other subsets, when sorted in isolation. Supplementation with anti-CTLA-4 or anti-PD-1 potentially could remedy the relative number of terminally differentiated tumor-specific CD8 T cells. We observed that bulk CD8 T cells were dominated by CD39^+^ cells with the majority co-expressing CD69 (**Figure 5D**). Within tumor-specific i.e. tetramer double positive CD8 T cells, we find that the majority were terminally differentiated (CD39^+^CD69^+^), which did not vary across TME checkpoint conditions (**Figures 5E, F**). Tumor-specific CD8 T cells, much like bulk CD8 T cells, therefore, exhibited unchanged frequency of terminally differentiated CD8 T cells. This is further underlined by the similarity between TME conditions observed in our metabolic assays. Multiple mechanisms downstream of PD-1 ligation have been identified that are understood to influence metabolic pathways which could alter the fate of cell determination (17, 41). We suspect the intervention with CPI was too late to reshape the bioenergetic profile of the TILs using anti-PD-1 and anti-CTLA-4 given the terminally differentiated state, which resulted in the retention of the metabolic footprint of the non-CPI TILs.

This study has demonstrated the ability to influence the specificity and proliferative capabilities of TILs through *ex vivo* modification of the microenvironment of tumor fragments. Our greatest limitation was the access to patient material. All samples were obtained from ongoing clinical trials at our center, which is currently the only way for patients with metastatic melanoma to receive TIL-based ACT. Therefore, all individuals had previously underwent treatment with, and had resistance to, one or more lines of immunotherapy. This inhibited our ability to evaluate to which extent prior treatment with CPI therapy influence the intact tumor fragments with anti-CTLA-4 and anti-PD-1 blocking antibodies. While we were unable to appreciate significant changes in phenotype or bioenergetics, we do demonstrate that TILs derived from patients with CPI-resistant metastatic melanoma retain a type of plasticity that can be influenced by disrupting PD-1 and CTLA-4 interactions.

## 4 Material and methods

### 4.1 Patient material

All patient material for this study was collected from a total of 15 patients with histologically verified malignant melanoma that underwent surgery between April 2016 and April 2018 throughout three different clinical trials at CCIT with an ECOG performance status of 0-1. All trials were approved by legal authorities. An overview of patient characteristics can be found in **Table 1**.

### 4.2 Generation of TIL cultures

Cultures were generated from tumor material that was obtained during resection in preparation for TIL-based ACT. Immediately after resection, fresh tumor tissue was transported to the cell laboratory, and manually fragmented. Wells of 24-well plates contained one fragment with 2 mL of complete medium (CM) consisting of RPMI1640 with GlutaMAX, 25 mM HEPES pH 7.2 (Gibco, 72400-021), 10% heat-inactivated human AB serum (HS, Sigma-Aldrich, H4522-100ML), 100 U/mL penicillin, 100 ug/mL streptomycin (Gibco, 15140-122), 1.25 µg/mL Fungizone (Bristol-Myers Squibb, 49182) and 6,000 IU/mL interleukin 2 (IL-2; proleukin, Novartis, 004184). The incubation conditions for all cultures throughout the study were 37°C at 5% CO_2_. Cultures were fed by replacing half of the medium three times weekly. For checkpoint inhibition-produced TILs, 5 µg/mL anti-CTLA-4 (Ipilimumab, Bristol-Myers Squibb) or 5 g/mL anti-PD-1 (Pembrolizumab, Merk/MSD) as indicated was added to the medium of initial TIL cultures and was supplemented with every media change. Eight fragments were used per condition. Initial TIL cultures were established *in vitro* according to the young TIL method by pooling TIL cultures derived from eight separate fragments (42). TIL cultures were considered established when more than 1 × 10^6^ cells/fragment could be generated within 35 days in one of the culture conditions yielding at least 8 x 10^6^ TILs per condition. All initial TILs from one patient across all conditions were harvested the same day in an effort to minimize culture time difference between conditions. Unestablished cultures generating less than 1 x10^6^ cells/fragment were harvested within three days of the other established conditions. For normalization, anti-CTLA-4 and anti-PD-1 group TILs per fragment counts were divided by the TILs per fragment count of the corresponding alone group for each patient.

### 4.3 Tumor cell cultures

Tumor cell lines were established either directly from tumor fragments, from media used for transportation of the tumor specimen or from enzymatically digested fresh tumor fragments. R10 medium consisting of RPMI1640 with GlutaMAX, 25 mM HEPES pH 7.2, 100 U/ml penicillin, 100 μg/ml streptomycin and 10% fetal bovine serum (FBS; Gibco, 10270-106) was used for culturing tumor cell lines.

### 4.4 Flow cytometry

#### 4.4.1 Phenotypic characterization of TILs

Cryopreserved TILs were stained for flow cytometry after thawing in RPMI1640 with GlutaMAX, 25 mM HEPES pH 7.2, 100 U/ml penicillin, 100 μg/ml streptomycin. Cells were washed in phosphate-buffered saline (PBS, Lonza, BE176-512F), stained at 4°C for 30 minutes, washed and resuspended in PBS. The cells were stained with Near-IR Live/Dead (NIR, Life Technologies, L10119). The fluorochrome-labeled monoclonal antibodies (from BD Biosciences, unless indicated otherwise) BV786 CD3 (563800), BV711 and PE-AF700 CD4 (563033 and MHCD0424, Invitrogen), PerCP-Cy5.5 and QDot605 CD8 (565310 and Q10009, Invitrogen), APC-R700 CD27 (565116), PE-Cy7 CD28 (560684), BV421 CD39 (563679), SB702 CD56 (67-0566-42, Life Technologies), PE-CF594 CD57 (562488), PE-Cy5.5 CD69 (MHCD6918, Invitrogen), BV421 B- and T-lymphocyte attenuator (BTLA, 564802), BV510 CCR7 (353232, Biolegend), BV570 CD45RO (304226, Biolegend), FITC lymphocyte-activation gene 3 (LAG-3, LS-B2237, LS Bioscience), PE-Cy7 PD-1 (561272), BV650 T-cell immunoglobulin and mucin-domain containing-3 (TIM-3, 565564) were used for surface staining. Subsequently, the cells were fixed and permeabilized (eBioscience, 00-5123-43, 00-5223-56 and 00-8333-56) at 4°C for 30 minutes and stained for intracellular cytokines PE TOX (thymocyte selection-associated high-mobility group box protein, 12-6502-82, Invitrogen) and AF647 TCF-1 (T-cell factor 1, 655204, Biolegend) and the stained cells were analyzed with a Novocyte Quanteon (ACEA Biosciences). The manufacturer information and clones of the flow cytometry antibodies are listed in **Supplementary Table S2**. Data analyses were carried out in Excel 2018 and GraphPad Prism 8. The change in the phenotypic subpopulations was investigated for statistical difference using Wilcoxon matched-pairs rank test. A two-sided P value of <0.05 was considered statistically significant.

#### 4.4.2 Evaluation of tumor reactivity

Anti-tumor reactivity of *in vitro* expanded TILs was evaluated after co-culture of the TILs with autologous or allogeneic tumor cell lines in a ratio of 3:1 (unless indicated otherwise) for 5 hours. All antibodies were purchased from from BD Biosciences, unless indicated otherwise. Golgi plug (BD Biosciences, 51-2301KZ) and BV421 CD107a (345812) were added at the beginning of incubation and TILs were stained with NIR and for surface markers BV786 CD3 (563800), SB702 CD56 (67-0566-42, Life Technologies), QDot605 CD8 (Q10009, Invitrogen), PerCP-Cy5.5 CD4 (317428, Biolegend), PE-Cy7 PD-1 (561272) and PE-CF594 CD39 (563678). Subsequently, the cells were fixed and permeabilized (00-5123-43, 00-5223-56 and 00-8333-56, eBioscience) overnight and stained for intracellular cytokines APC TNF (554514) and BV510 IFN-γ (502544, Nordic Biosite). The manufacturer information and clones of the flow cytometry antibodies are listed in **Supplementary Table S3**. The TILs were analyzed with a Novocyte Quanteon. Tumor-reactive TILs were defined as T cells expressing either TNF, IFN-γ or CD107a. The response in an unstimulated sample (negative control) was subtracted from the stimulated samples.

### 4.5 Measurements of bioenergetics

The bioenergetics from patient-derived TILs was measured in real-time using an XF-96 Extracellular Flux Analyzer (Seahorse Bioscience, Agilent). On day one, Cryopreserved TILs were thawed and rested overnight in RPMI-1640 (Gibco, Ref: 72400-021) supplemented with 10% heat-inactivated human AB serum and penicillin/streptomycin. On day two, the media was supplemented with IL-2 (6000 IU/mL), and the TILs were left in culture for an additional two days. On day four, the TILs were collected and resuspended in Seahorse Assay Media (Seahorse Bioscience, Agilent) supplemented with 1.0 mmol/L sodium pyruvate (Agilent, Cat: 103578-100), 3.0 mmol/L glutamine (Agilent, Cat: 103579-100), and 4.0 mmol/L glucose (Cat: 103577-100). The TILs were then plated in a Seahorse 96-well plate (300.000 cells/well). Oxygen consumption rates (OCR) and extracellular acidification rates (ECAR) were measured as the wells were consecutively treated with 0.75 µM oligomycin (Sigma), 0,4 µM carbonyl cyanide p-(trifluoromethoxy) phenylhydrazone (FCCP), and 2 µM Antimycin A (Sigma). After the Extracellular Flux assay, the plate was washed once in PBS (Sigma, ref: D8537) and the cells were then resuspended in RIPA buffer (ThermoScientific, ref: 89900) and centrifuged again. To determine the protein concentration in each well in the extracellular flux assay, the supernatants were then collected and used in a BCA protein assay kit (ThermoScientific, ref: 23225) according to the manufacturer’s instructions. Results were analysed using the Epoch plate reader (BioTek) and Gen5 Take3 software (v1.00.4 BioTek) and were performed to normalize the OCR and ECAR measurements according to the protein content.

### 4.6 Evaluation of specificity for shared tumor antigens

#### 4.6.1 Shared tumor antigens

A library of 156 unique HLA-A2-restricted tumor antigens was used to screen TILs from patients with metastatic melanoma for recognition of HLA-A2-restricted tumor-associated CD8^+^ T-cell epitopes (**Supplementary Table S1**). The tumor antigen peptide library can be grouped into four classes of antigens; differentiation antigens: 26, cancer-testis antigens: 44, overexpressed antigens: 78 and mutation antigens: 6.

#### 4.6.2 HLA typing

Whole exome (WXS) and RNA sequencing were performed as described previously (43). Briefly, a fragment of each tumor used for TIL production was cryopreserved and used for WXS. Mononuclear cells were used as matched normal sample. DNA and RNA were extracted from tumors and normal samples using AllPrep DNA/RNA Mini Kit (Qiagen). WXS libraries were prepared using SureSelect Target Enrichment System for Illumina Paired-End Sequencing Library Protocol with Clinical Research Exome (CRE) capture panel (Agilent Technologies) and sequenced on NextSeq500 (Illumina). For HLA typing, the trimmed WXS data were analyzed with OptiType version 1.2 (44) using RazerS version 3.4.0 (45) to align the reads. The trimmed RNA sequencing reads were used as input to Kallisto 0.42.1 (46) to find the expression per transcript. All peptides were purchased from Pepscan (Pepscan Presto BV, Lelystad, Netherlands) and dissolved to 10 mM in DMSO. A full list of TAA-derived peptides and virus peptides in the study can be found in can be found in **Supplementary Tables S1, S4**, respectively.

#### 4.6.3 MHC monomer production and generation of specific pMHC multimers

The production of MHC monomers was performed as previously described by Hadrup et al. (47). In brief, HLA heavy chains and human β_2_m light chain were expressed in bacterial BL21 (DE3) pLysS strain (Novagen, Cat# 69451) and purified as inclusion bodies. After solubilization, HLA inclusion bodies were refolded with β_2_m light chain and a UV-sensitive ligand (48, 49) and the folded monomers were biotinylated with BirA biotin-protein ligase standard reaction kit (Avidity, 318 LLC-Aurora, Colorado) and purified using a size-exclusion column (Waters, BioSuite125, 13µm SEC 21.5 × 300 mm) and HPLC (Waters 2489).

#### 4.6.4 Detection of peptide-MHC-specific T cells by barcoding

DNA-barcoded pMHC multimers were used to screen for T cell recognition of all tumor- and virus derived epitopes. The method is described in detail in Bentzen et al. (50). Briefly, peptide loading of empty monobiotinylated A*02:01 monomers was performed. Each generated pMHC complex was coupled to DNA barcode- and PE- or APC-labeled dextran backbones (Fina Biosolutions LLC), so that each specific peptide was encoded by a unique DNA barcode. Metastatic melanoma TILs were stained with a pool of all barcoded MHC-multimers and an antibody mix of CD8-BV480 (566121, BD), dump channel antibodies (CD4-FITC (345768, BD), CD14-FITC (345784, BD), CD19-FITC (345776, BD), CD40-FITC (MCA1590F, Bio-Rad), and CD16-FITC (335035, BD), and the dead cell marker LIVE/DEAD Fixable Near-IR. The manufacturer information and clones of the flow cytometry antibodies are listed in **Supplementary Table S5**. Multimer-specific T cells were then sorted as single, live, CD8^+^, FITC^-^, PE^+^ or APC^+^ fraction of cells, pelleted by centrifugation and cryopreserved at -80°C. DNA barcodes were amplified from the cell pellet and from a stored aliquot of the pMHC multimer reagent pool (used as baseline for comparison) by PCR, purified with a QIAquick PCR Purification kit (Qiagen, Cat#28104) and sequenced (Primbio, USA) using an Ion Torrent PGM 318 chip (Life Technologies). Sequencing data were processed by the software package “Barracoda”, available online at http://www.cbs.dtu.dk/services/barracoda. This tool identifies barcodes used in a given experiment, assigns sample ID and pMHC specificity to each barcode, and counts the total number (clonally reduced) reads for each pMHC-associated DNA barcode. Log_2_ fold changes in read counts mapped to a given sample relative to the mean read counts mapped to triplicate baseline samples are estimated with normalization factors determined by the trimmed mean of M-values method. False-discovery rates (FDRs) were estimated using the Benjamini–Hochberg method. A P value is calculated based on the Log_2_ fold change distribution, determining the strength of the signal compared to the input. Also, *p* < 0.001, corresponding to FDR < 0.1%, is established as the significance level determining a T-cell response. Frequency of a pMHC-specific CD8^+^ T cell (sum est freq) was calculated as the fraction of the associated barcode of the total number of multimer-positive CD8^+^ T cells.

#### 4.6.5 Validation of tumor antigen specific CD8^+^ T cells using combinatorial-encoding of fluorescence tetramers

Combinatorial encoding of fluorescent tetramers was performed as described previously (27, 28) with the following adaptations: Streptavidins were bought fluorescently conjugated to BUV737 (612775, BD), BV421 (563295, BD), BV650 (563855, BD), BV786 (563858, BD), PE (405204, Biolegend), PE-CF594 (562318, BD), PE-Cy7 (557598, BD) and APC (105243, Biolegend). The following combinations of tetramers were regarded as viable unique identifiers of a response with manageable fluorescent spillover: APC x PE-CF594, APC x PE, APC x PE-Cy7, APC x BV421, APC x BV786, APC x BUV737, PE-CF594 x PE, PE-CF594 x PE-Cy7, PE-CF594 x BV421, PE-CF594 x BV786, PE-CF594 x BUV737, PE-CF594 x BV650, PE x PE-Cy7, PE x BV421, PE x BV786, PE x BUV737, PE x BV650, PE-Cy7 x BV421, PE-Cy7 x BV786, BV421 x BV786, BV421 x BUV737. Streptavidins combinations were premixed at differing ratios and stored at 4*C until multimer assembly. Any PE or APC combined with the dimmer BV or BUV colors were mixed in ratio of 1:2, whereas all remaining combinations were premixed 1:1. Streptavin concentration in the final mix was 0.1 mg/ml. Following peptide loading at, final concentration, 100 µg/ml pMHC both streptavidin mixes and loaded pMHC complexes were spun at 3300 g, 4°C, 5 mins before addition of 25.5 µl pMHC supernatant [100 µg/ml] to 4.63 µl tetramer supernatant [0.1 mg/ml]. Loaded streptavidins were then incubated for 30 minutes at 4°C before addition of freezing buffer containing 50% glycerol, 5% BSA and 173 µM D-Biotin to a final concentration of 24.1 µM D-Biotin followed by incubation for 20 minutes at 4°C before freezing and storage at -21°C.

Staining of tetramer specific CD8 T cells was performed before staining with surface antibodies by hard centrifugation of tetramer combinations 3 times at 3300g, 4°C, 5 mins with transfers between spins to separate wells without pellets. Patient-specific pools of tetramers were then defined and used for resuspension of young TIL cultures pre-washed in FACS buffer (2% FCS). Staining was performed with 1 µl per tetramer combination in 50 µl total volume. Dasatinib was added along with tetramers at a final concentration of 100 nM. Surface antibodies were added subsequently and included CD3-PerCP (300427, Biolegend), CD4-FITC (347413, BD), CD8-BV480 (566121, BD), CD69-BUV395 (564364, BD) and CD39-FITC (328205, Biolegend). Pregating was performed on CD3^+^CD4^-^CD8^+^ T cells with tetramers positive for exactly the combination of interest and not for any of the other streptavidins used. Excluded specificities included LVHFLLLKY-MAGE-A3, SLLMWITQA-NY-ESO-1 and SLGWLFLLL-TAG-1 due to failed tetramer assembly (i.e. strong pellet formation during staining or empty peptide aliquots).

### 4.7 Statistics

Data analyses were carried out in Excel 2018 and GraphPad Prism 8. The change in the TIL expansion, phenotypic subpopulations, specificity and tumor reactivity was investigated for the statistical difference using Wilcoxon matched-pairs rank test. A two-sided P value of <0.05 was considered statistically significant. For TAA-specificity validation experiments, statistical analysis was performed using unpaired kruskal-wallis test to access overall differences between expansion conditions. Rstudio (2022.07.1) was used with R 4.0.5 to calculate P values and generate plots by use of the tidyverse package suite (51) cowplot.

## Data availability statement

The datasets presented in this article are not readily available because the Danish Health Law “Sundhedsloven” and Personal Data Protection Law “Persondataloven,” does not permit the deposition of individual patient data to public repositories. Requests to access the datasets should be directed to the senior authors who may review and release all available data directly to the external researcher via a data processing agreement that must be approved by the Knowledge Centre on Data Protection Compliance, Capital Region of Denmark.

## Ethics statement

The studies involving human participants were reviewed and approved by National Videnskabsetisk Komité. The patients/participants provided their written informed consent to participate in this study.

## Author contributions

TH and ÖM wrote the manuscript. TH, NK, CF and JG carried out experiments and performed data analysis. ÖM conceptualized and supervised the study. All authors contributed to the article and approved the submitted version. 
