# Corrigendum: *Ex vivo* modulation of intact tumor fragments with anti-PD-1 and anti-CTLA-4 influences the expansion and specificity of tumor-infiltrating lymphocytes

**DOI:** 10.3389/fimmu.2024.1462081

**Published:** 2024-07-18

**Authors:** Thomas Morgan Hulen, Christina Friese, Nikolaj Pagh Kristensen, Joachim Stoltenborg Granhøj, Troels Holz Borch, Marlies J. W. Peeters, Marco Donia, Mads Hald Andersen, Sine Reker Hadrup, Inge Marie Svane, Özcan Met

**Affiliations:** ^1^ National Center for Cancer Immune Therapy (CCIT-DK), Department of Oncology, Copenhagen University Hospital, Herlev, Denmark; ^2^ Department of Health Technology, Technical University of Denmark, Lyngby, Denmark

**Keywords:** tumor-infiltrating lymphocyte (TIL), checkpoint inhibition, metastatic melanoma, cancer immunotherapy, tumor microenevironment, adoptive cell immunotherapy, DNA Barcoding

In the published article, there was an error in **Figure 4** as published. Antigen category of “Overexpressed Antigens” was mislabeled. The corrected **Figure 4** and its caption appear below.

**Figure 4:**
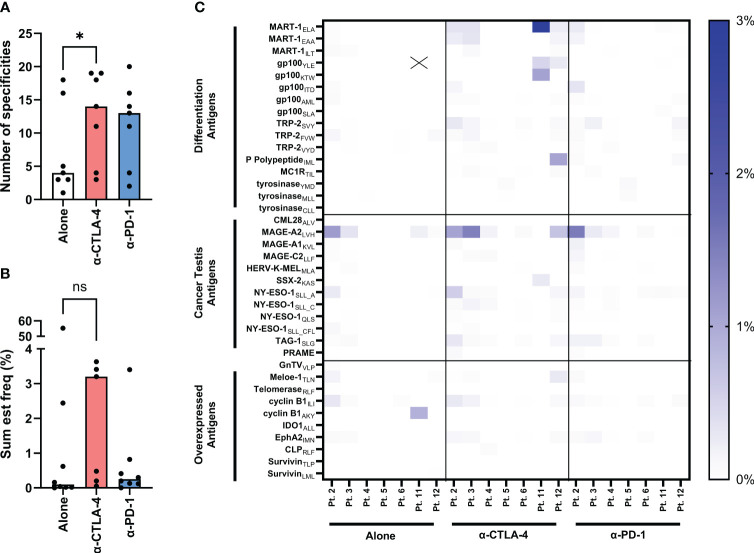
Evaluation of tumor-specificities of yTILs produced with IL-2 alone or CPI. yTILs were stained with pooled tetramers loaded with 156 unique HLA-A2-restricted epitopes. **(A)** shows the number of tumor antigens yTILs from each patient had across conditions with anti-CTLA-4 group having statistically significant more specificities than alone (P value = 0.0156), while the anti-PD-1 group is not significant (P value = 0.938). **(B)** shows the sum of the estimated frequencies of tumor antigens across conditions. The sum est freq of the anti-CTLA-4 group is not statistically significant (P value = 0.2969). The individual specificity frequencies for each tumor antigen across 3 antigen categories are represented with a heatmap **(C)**. “X” indicates value of 54.17%. Wilcoxon test was applied to compare paired data sets. “ns” = not significant; “*” represents the p value which is listed in the image description.

In the published article, there was also an error in **Table 2** as published. Column heading of “%CD8+ of CD3+” was mislabeled and English decimal, “.” was not used. The corrected **Table 2** and its caption appear below.

**Table 2:** Sample overview including TILs/fragment, CD4 and CD8 frequencies, and ICS tumor stimulation for the three TME conditions.

Patient #	CPI Modulation	TILs/Fragment (x10^6)	%CD4+ of CD3+	%CD8+ of CD3+	ICS Tumor Stimulation
MM 1	Alone	3.66	31.2	66.2	Allogenic
	Anti-CTLA-4	2.63	29.2	65.7	
	Anti-PD-1	2.00	15.5	80.6	
MM 2	Alone	4.23	47.1	47.3	Allogenic
	Anti-CTLA-4	4.23	58.1	37.2	
	Anti-PD-1	6.21	70.8	25.1	
MM 3	Alone	3.60	37.7	48.8	Allogenic
	Anti-CTLA-4	7.43	26.7	63.2	
	Anti-PD-1	3.93	31.0	24.4	
MM 4	Alone	0.50	27.5	69.3	Allogenic
	Anti-CTLA-4	0.74	39.4	56.1	
	Anti-PD-1	8.00	7.40	91.0	
MM 5	Alone	5.18	52.0	40.7	Autologous
	Anti-CTLA-4	2.78	87.2	10.8	
	Anti-PD-1	3.65	61.6	35.1	
MM 6	Alone	5.14	87.9	11.1	Allogenic
	Anti-CTLA-4	2.60	7.90	83.7	
	Anti-PD-1	4.30	48.6	49.3	
MM 7	Alone	0.23	48.1	47.4	Allogenic
	Anti-CTLA-4	0.09	46.0	44.9	
	Anti-PD-1	0.95	22.6	68.4	
MM 8	Alone	4.15	21.6	75.6	Allogenic
	Anti-CTLA-4	2,73	72.9	24.9	
	Anti-PD-1	4.35	26.8	69.9	
MM 9	Alone	5.25	18.7	79.6	Autologous
	Anti-CTLA-4	2.55	11.8	84.9	
	Anti-PD-1	2.70	33.2	63.0	
MM 10	Alone	5.16	8.90	89.7	Autologous
	Anti-CTLA-4	6.01	12.7	85.9	
	Anti-PD-1	6.23	11.0	87.4	
MM 11	Alone	11.18	69.1	29.3	Autologous
	Anti-CTLA-4	1.78	35.3	56.0	
	Anti-PD-1	16.2	2.00	97.1	
MM 12	Alone	1.76	28.0	68.0	Autologous
	Anti-CTLA-4	3.44	9.80	83.0	
	Anti-PD-1	5.41	17.2	77.5	
MM 13	Alone	0.03	57.2	33.2	n.d.
	Anti-CTLA-4	0.30	23.8	69.3	
	Anti-PD-1	17.43	6.70	91.9	
MM 14	Alone	2.98	39.2	59.2	Allogenic
	Anti-CTLA-4	2.69	50.5	43.6	
	Anti-PD-1	6.94	78.2	19.1	
MM 15	Alone	n.d.	n.d.	n.d.	n.d.
	Anti-CTLA-4	n.d.	n.d.	n.d.	
	Anti-PD-1	n.d.	n.d.	n.d.	

Red text indicates culture not established. N.d. indicates no data due to lack of TIL production.

The authors apologize for these errors and state that they do not change the scientific conclusions of the article in any way. The original article has been updated.

