# Supplementary material for: Ex vivo modulation of intact tumor fragments with anti-PD-1 and anti-CTLA-4 influences the expansion and specificity of tumor-infiltrating lymphocytes
Source: Front Immunol. 2023 Jun 8;14:1180997. doi: 10.3389/fimmu.2023.1180997 (PMC10285209; doi:10.3389/fimmu.2023.1180997)
Supplement: Supplementary file 1 [file DataSheet_1.docx]

Supplementary Material

# Supplementary Figures

## Figure S1

**Supplementary Figure S1**: Exhaustion markers of CD4+ and CD8+ TILs across all three conditions. Addition of CPI to the intact tumor fragments result in no notable difference between these conditions, with the exception of PD-1 expression. Binding of the staining antibody is disrupted by the PD-1 blocking antibody used in production.

## Figure S2

**Supplementary Figure S2:** Fold change during rapid expansion protocol (REP). TILs from unmodulated or anti-PD-1 modulated tumor fragments underwent REP in the presence or absence of anti-PD-1. “-” indicates no checkpoint inhibitor, “P” indicates anti-PD-1.

## Figure S3


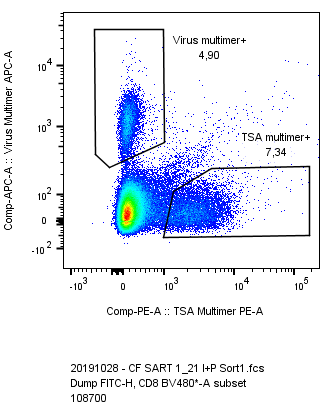


PE (TAA multimer)

APC (virus multimer)

**A**

**Supplementary Figure S3:** Representative tetramer stain FACS plot. Tumor antigens linked to PE multimers, while virus antigens linked to APC multimers. Gated populations subsequently sorted for amplicon sequencing.

## Figure S4

**Supplementary Figure S4:** Summary of the 20 antigen specificities of highest detection within TIL cultures

## Figure S5

**Supplementary Figure S5:** Summary of the 20 highest sum est freq of tumor specicity within TIL cultures

## Figure S6

**Supplementary Figure S6:** Summary of viral antigen specificities detected by multimer staining **.**

## Figure S7

**Supplementary Figure S7:** Sum est freq of all viral antigen specificities detected within TIL cultures.

## Figure S8

### Figure S8.1


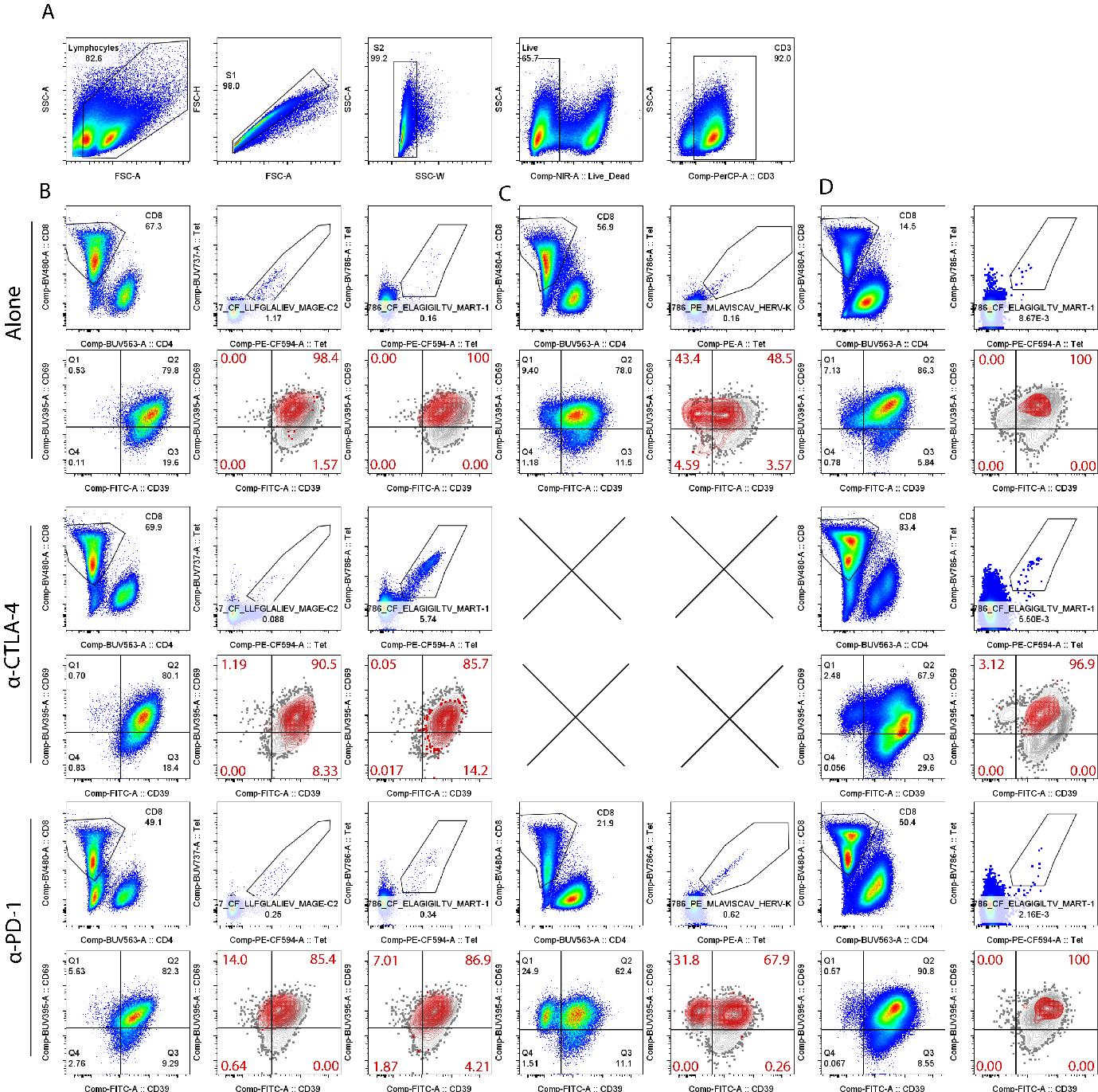


**Supplementary Figure S8.1:** Tumor-specific CD8 T cells. **(A)** Gating strategy for isolation of tetramer+ CD8 T cells. **(B-D)** Frequency and CD39+CD69+ phenotype of individual tumor-specific populations (red) and bulk CD8 T cells (grey). Expansion conditions indicated for each row. **(B)** Data for MM 3, **(C)** Data for MM 5, **(D)** Data for MM 6. ”X” marks conditions that were excluded prior to staining due to lacking donor material.

### Figure S8.2


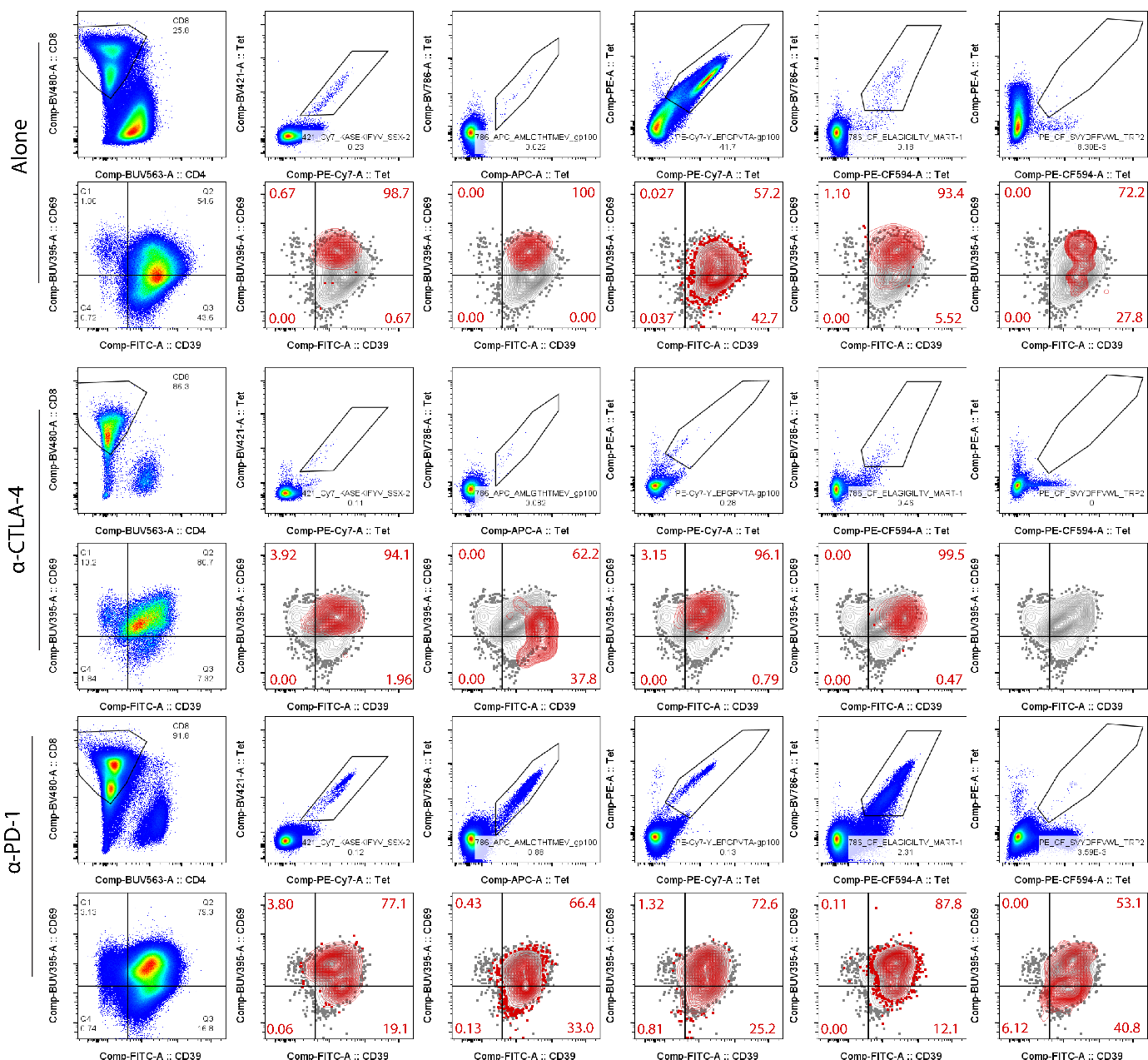


**Supplementary Figure S8.2:** Tumor-specific CD8 T cells and their CD39+CD69+ phenotype (red) and bulk CD8 T cells (grey). Data for MM 11. Expansion conditions indicated for each row.

### Figure S8.3


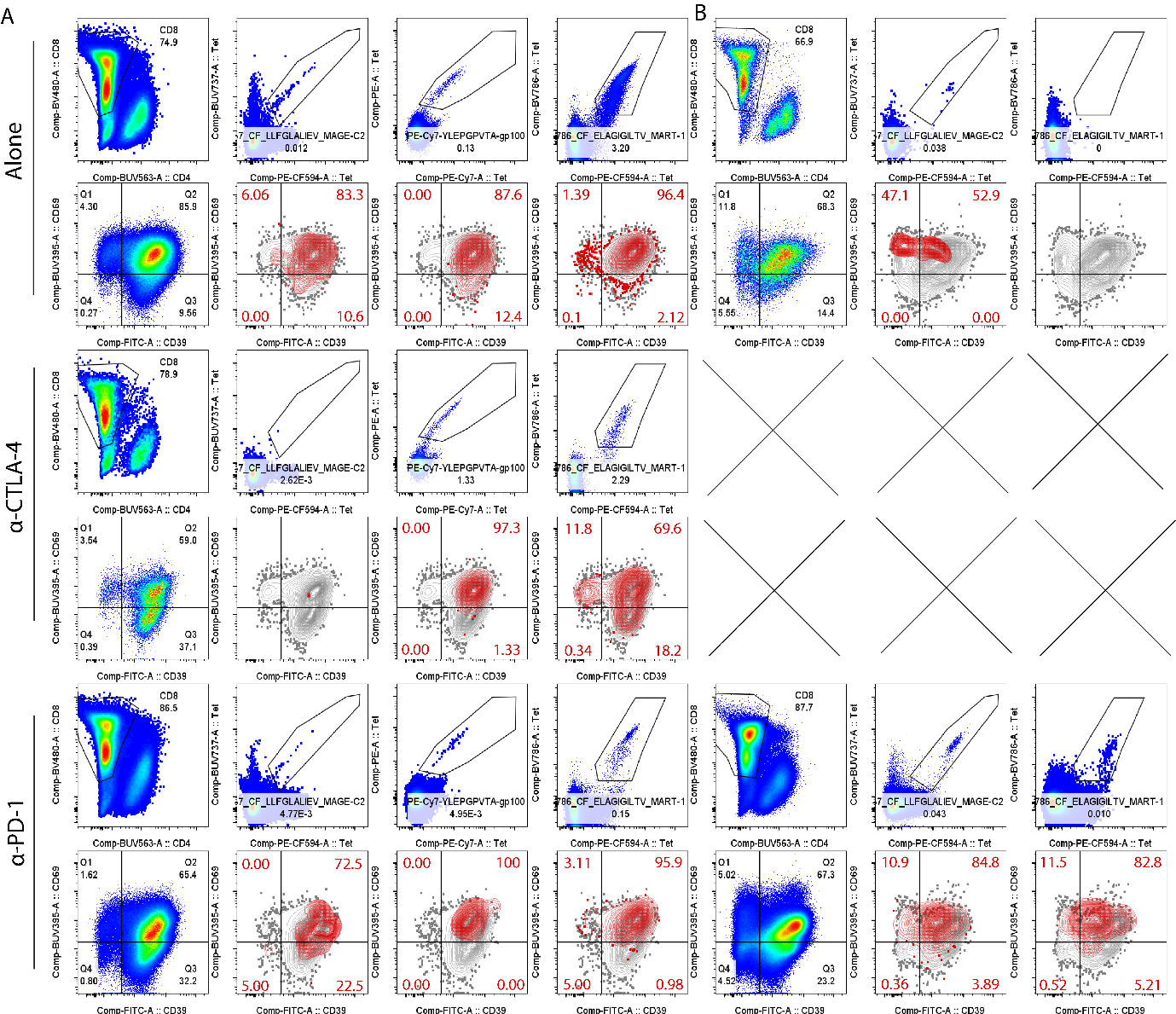


**Supplementary figure S8.3:** Tumor-specific CD8 T cells and their CD39+CD69+ phenotype (red) and bulk CD8 T cells (grey **(A)** Data for MM1414.13. **(B)** Data for MM 4. Note that the single MAGE-C2 specific event recorded in the anti-CTLA-4 condition was excluded from further phenotyping of CD39+CD69+. Expansion conditions indicated for each row. ”X” marks conditions that were excluded prior to staining due to lacking donor material.

# Supplementary Tables

##
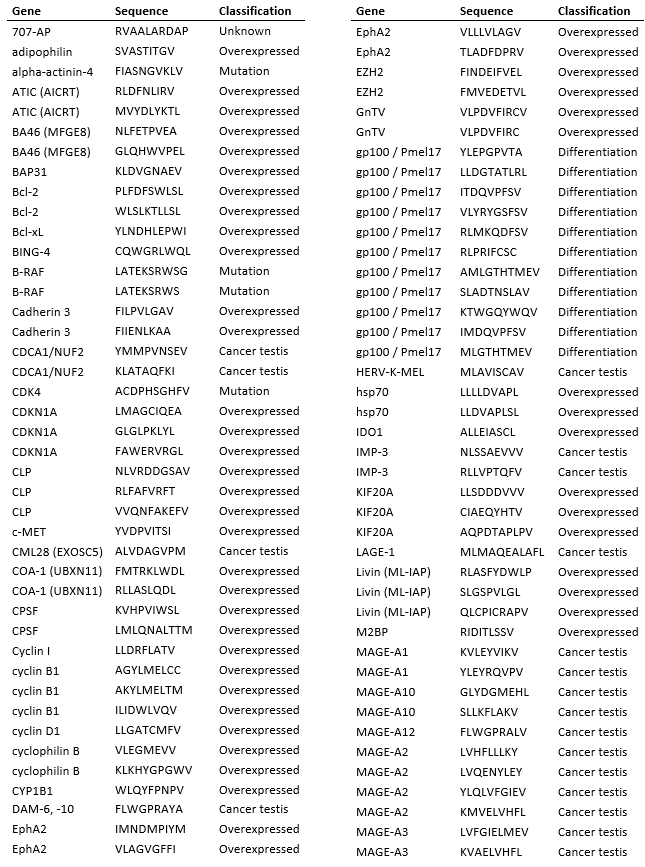
Supplementary Table S1: Tumor-antigens included in TIL screen.


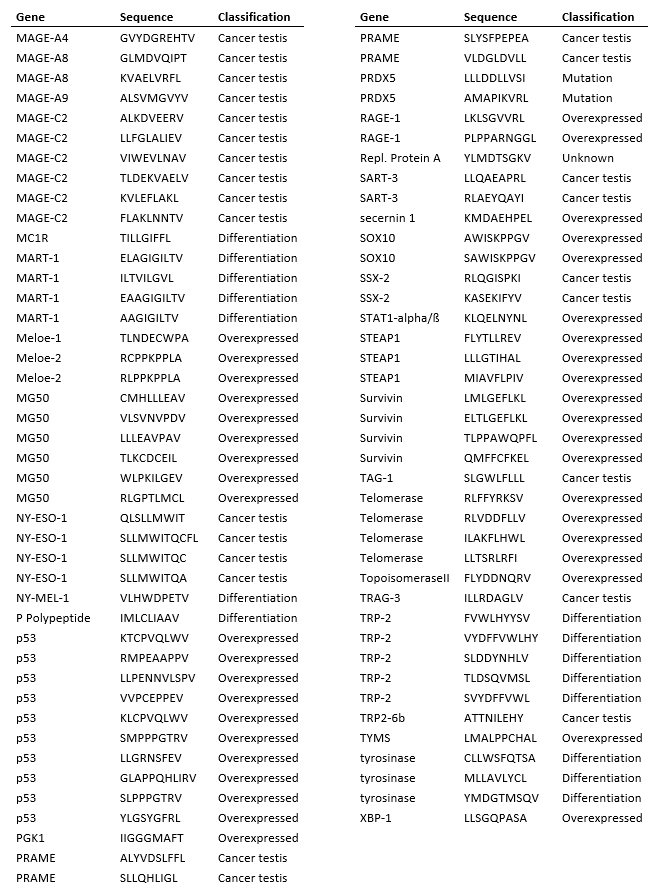


## Supplementary Table S2: Flow cytometry antibodies used for phenotyping.

| Antigen | Fluorochrome | Clone | Manufacturer | Catalog No. |
| --- | --- | --- | --- | --- |
| BTLA | BV421 | J168-540 | BD | 564802 |
| CCR7 | BV510 | G043H7 | Biolegend | 353232 |
| CD3 | BV786 | SK7 | BD | 563800 |
| CD4 | PE-AF700 | S3.5 | Invitrogen | MHCD0424 |
|  | BV711 | SK3 | BD | 563033 |
| CD8 | PerCP-Cy5.5 | SK1 | BD | 565310 |
|  | QDot605 | 3B5 | Invitrogen | Q10009 |
| CD27 | APC-R700 | M-T271 | BD | 565116 |
| CD28 | PE-Cy7 | CD28.2 | BD | 560684 |
| CD39 | BV421 | TU66 | BD | 563679 |
| CD45RO | BV570 | UCHL1 | Biolegend | 304226 |
| CD56 | SB702 | TULY56 | Life Technologies | 67-0566-42 |
| CD57 | PE-CF594 | NK-1 | BD | 562488 |
| CD69 | PE-Cy5.5 | CH/4 | Invitrogen | MHCD6918 |
| LAG-3 | FITC | 17B4 | LS Bioscience | LS-B2237 |
| PD-1 | PE-Cy7 | EH12.1 | BD | 561272 |
| TIM-3 | BV650 | 7D3 | BD | 565564 |

## Supplementary Table S3: Flow cytometry antibodies used for tumor-reactivity analysis.

| Antigen | Fluorochrome | Clone | Manufacturer | Catalog No. |
| --- | --- | --- | --- | --- |
| CD3 | BV786 | SK7 | BD | 563800 |
| CD4 | PerCP-Cy5.5 | OKT4 | Biolegend | 317428 |
| CD8 | QDot605 | 3B5 | Invitrogen | Q10009 |
| CD56 | SB702 | TULY56 | Life Technologies | 67-0566-42 |
| CD107a | BV421 | H4A3 | BD | 345812 |
| IFN-γ | BV510 | 4S.B3 | Biolegend | 502544 |
| TNF | APC | Mab11 | BD | 554514 |

## Supplementary Table S4: Virus-derived peptides included in TIL screening.

| **Virus** | **HLA** | **Sequence** |
| --- | --- | --- |
| **Cytomegalovirus** | HLA-A01:01 | YSEHPTFTSQY |
|  | HLA-A02:01 | NLVPMVATV |
|  | HLA-A01:01 | VTEHDTLLY |
|  | HLA-A02:01 | VLEETSVML |
|  | HLA-B44:02 | EFFWDANDI |
| **Epstein Barr Virus** | HLA-A02:01 | CLGGLLTMV |
|  | HLA-A02:01 | GLCTLVAML |
|  | HLA-A02:01 | FLYALALLL |
|  | HLA-A02:01 | YVLDHLIVV |
|  | HLA-B44:02 | EENLLDFVRF |
| **Human immunodeficiency virus** | HLA-A02:01 | ILKEPVHGV |
| **Influenza** | HLA-A02:01 | GILGFVFTL |
|  | HLA-A01:01 | VSDGGPNLY |

## Supplementary Table S5: Flow cytometry antibodies used for sorting of tumor-antigen specific T cells.

| Antigen | Fluorochrome | Clone | Manufacturer | Catalog No. |
| --- | --- | --- | --- | --- |
| CD4 | FITC | SK3 | BD | 345768 |
| CD8 | BV480 | RPA-T8 | BD | 566121 |
| CD14 | FITC | MφP9 | BD | 345784 |
| CD16 | FITC | NKP15 | BD | 335035 |
| CD19 | FITC | 4G7 | BD | 345776 |
| CD39 | BV421 | TU66 | BD | 563679 |
| CD40 | FITC | LOB7/6 | Bio-Rad | MCA1590F |
| PD-1 | PE-Cy7 | EH12.1 | BD | 561272 |
